# Heterogeneity in Health Outcomes in the Strong Hearts, Healthy Communities-2.0 Multilevel Intervention in a Community-Randomized Trial: An Exploratory Study of Moderators

**DOI:** 10.3390/nu16244353

**Published:** 2024-12-17

**Authors:** Chad D. Rethorst, Margaret M. Demment, Seungyeon Ha, Sara C. Folta, Meredith L. Graham, Galen D. Eldridge, Rebecca A. Seguin-Fowler

**Affiliations:** 1 Institute for Advancing Health Through Agriculture, Texas A&M University, Dallas, TX 75252, USA; chad.rethorst@ag.tamu.edu (C.D.R.); margaret.demment@ag.tamu.edu (M.M.D.); meredith.graham@ag.tamu.edu (M.L.G.); galen.eldridge@ag.tamu.edu (G.D.E.); 2 Statistical Consultation Center, Texas A&M University, College Station, TX 77843, USA; ha.shawn@tamu.edu; 3 Friedman School of Nutrition Science and Policy, Tufts University, Boston, MA 02111, USA; sara.folta@tufts.edu; 4 Institute for Advancing Health Through Agriculture, Texas A&M University, College Station, TX 77840, USA

**Keywords:** obesity, cardiovascular disease, multilevel intervention, moderation analysis, diet, nutrition, physical activity, mental health

## Abstract

Background/Objectives: Multilevel interventions have demonstrated efficacy in improving obesity and other related health outcomes. However, heterogeneity in individual responses indicates the need to identify the factors associated with responses and non-responses to multilevel interventions. The objective of this report is to identify the potential sources of heterogeneity through the exploration of the moderation effects of participant characteristics (sociodemographic and baseline physical/mental health) in the Strong Hearts, Healthy Communities-2.0 (SHHC-2.0) intervention. Methods: SHHC-2.0 is a 24-week multilevel intervention to improve people’s diet and physical activity evaluated using a cluster-randomized, controlled trial design conducted with women aged 40 and older living in rural communities with an elevated risk of cardiovascular disease, defined as having a BMI > 30, or a BMI 25–30 plus < 1 weekly occurrence of 30 min of physical activity during leisure time. Linear mixed models were used to compare the between-group changes in the outcomes (weight, systolic blood pressure, hemoglobin A1c [HbA1c], and triglycerides), with an interaction term included for each potential moderator. Results: Within the sociodemographic characteristics, there were no differences in effectiveness by age, income, or baseline BMI status, however the participants with a high school education or less experienced greater weight loss. Among their health history, only a history of hypertension was associated with differential outcomes; those with a history of hypertension demonstrated a greater reduction in systolic blood pressure. The participants with elevated depressive symptoms demonstrated greater weight loss and a greater reduction in the HbA1c level. Conclusions: SHHC-2.0 was effective across a wide range of participants. The identified moderators (i.e., education level) may inform the future tailoring of the SHHC intervention to optimize the outcomes among participant subgroups, while more broadly, our findings can serve to inform the development and dissemination of multilevel interventions.

## 1. Introduction

Obesity contributes to poor health outcomes [1], a lower quality of life [2], and higher healthcare costs [3]; the direct medical costs due to obesity in adults in the United States are more than USD 260.6 billion per year [3]. The successful behavior change interventions for obesity typically include increased physical activity, improved diet quality, goal setting, and self-monitoring [4]. These types of intervention can result in weight loss averaging 8 kg or 5–10% of their body weight [4,5]. Participants also often improve their cardiovascular disease risk factors (e.g., hemoglobin A1c [HbA1c], blood pressure, and triglycerides) and quality-of-life measures (e.g., improvements in physical function, self-esteem, and psychosocial function and reduced symptoms of depression) [4].

Due to the complex causes of obesity, multilevel interventions that aim to change behaviors and address health outcomes by affecting more than one level of influence within the socioecological model are recommended [6]. Over the last two decades, multilevel interventions to address obesity have expanded greatly, and the results are promising, with most multilevel interventions improving the measures of adiposity (e.g., body weight, body mass index [BMI], and waist circumference) and/or obesity-related behaviors (e.g., physical activity and diet) [6]. Despite the significant benefits of behavior change interventions on obesity and other health outcomes, many studies result in the significant heterogeneity in outcomes [7]. The heterogeneity in treatment responses refers to the idea that the true effect of a treatment on one individual will be different than the effect on a different individual [8].

There are many potential sources of the heterogeneity in treatment responses beyond adherence to the intervention, including complex biological reasons (e.g., resting energy requirements based on metabolic programming and the impact of gastrointestinal peptides and the gut biome); the bi-directional relationship between mental health and weight loss; and the social determinants of health (e.g., having had access to high-quality education; economic stability; the social context; and the built environment). Detailing these factors will allow for more accurate predictions of intervention success and more effectively tailored strategies [9]. The analysis of 32 adult obesity studies found that 12 of them showed evidence that treatment heterogeneity was present, and of those 12, 3 demonstrated an estimated proportion of >5% of the sampled population having an outcome opposite to the mean effect (e.g., weight gain instead of weight loss) [8].

In a recent review of multilevel interventions targeting obesity, fewer than one in ten interventions reported differences in the outcomes across sociodemographic factors, and those that did showed differences in effectiveness by race, ethnicity, obesity status, age, and sex [6]. Much remains to be understood about which individuals benefit from lifestyle behavior interventions, with critical implications for dissemination and addressing health equity. Understanding who benefits the most (or least) from an intervention is explored through moderator analyses [10]. Strong Hearts, Healthy Communities-2.0 (SHHC-2.0) is a multilevel intervention that aimed to reduce the cardiovascular disease risk among rural women through targeting behavioral determinants at the individual, social, and environmental levels. SHHC-2.0 has demonstrated improvements in cardiovascular disease risk, [11] that were maintained at a 6-month follow-up [12], while increasing physical activity levels [13] and improving diet quality [13,14]. This report’s objective is to explore the moderation effects of the sociodemographic and baseline physical and mental health characteristics of SHHC-2.0 participants on selected health outcomes (weight, systolic blood pressure, HbA1c, and triglycerides).

## 2. Methods

This community-randomized control trial was conducted between January 2017 and August 2018. Eleven rural, medically underserved communities in New York were pair-matched based on the Rural–Urban Commuting Area (RUCA) code and population size, and then randomized into the SHHC-2.0 intervention or a delayed-intervention control condition, in which intervention delivery followed a 24-week primary outcome period. The participants were female and >40 years old at enrollment and were required to have a BMI > 30, or a BMI 25–30 plus < 1 weekly occurrence of 30 min of leisure-time physical activity. A full description of the study design and related effectiveness reports have been published elsewhere [11,12].

### 2.1. Intervention

The SHHC-2.0 intervention included 60-min classes delivered two times per week for 24 weeks, led by local health educators. The intervention was built around goals and behavioral aims for both diet and physical activity. Specifically, the physical activity behavioral aims included increasing strength training to two or more times per week, increasing aerobic activity to at least five times per week, and to minimize sedentary time. The dietary aims included eating fewer calories, saturated and trans fats, processed foods, desserts, and sugar-sweetened beverages and less sodium and increasing fruit, vegetable, and whole grain consumption. The social environment’s influence on diet and exercise behavior was engaged and reinforced through concepts such as social support for healthy behaviors; healthy eating plans for friends and family; social influences on sugar-sweetened beverage consumption; and what to do when loved ones are unsupportive. The intervention’s Change Clubs engaged participants to identify an issue relevant to them in their community context and facilitate the implementation of an action plan that would affect the social, cultural, environmental, and political factors within each of their communities.

### 2.2. Measures

At baseline and the end of the intervention (24 weeks), the participants completed a data collection visit that included anthropometric and biometric measurements (venipuncture, weight, height, and blood pressure), functional fitness tests, and a survey of self-reported behaviors and psychosocial variables.

#### 2.2.1. Outcomes

The primary outcome for this study was change in body weight; the secondary outcomes included herein due to an observed intervention effect were systolic blood pressure, triglycerides, and HbA1c. The equipment used included Omron (Kyoto, Japan) HBF-510W scales for body weight and Omron automated devices for blood pressure. Fasting blood specimens were collected by phlebotomists and registered nurses to measure triglycerides and HbA1c.

#### 2.2.2. Moderators

Baseline measures were used as the potential moderators for sociodemographic characteristics, physical health, and mental health. The sociodemographic characteristics included the following: age, employment status, household income (<USD 50,000 versus ≥ USD 50,000), education (some college or higher versus high school or less), and relationship status (yes or no). The SHHC-2.0 participants were predominantly White, non-Hispanic (97.6%); therefore, no moderation was explored by race or ethnicity. The potential baseline physical health moderators included measured BMI; the Short Form 36 (SF36) health-related quality-of-life survey scale [15]; and a self-reported history of arthritis, cancer, diabetes, heart disease, high blood cholesterol levels, high blood sugar levels, or hypertension. The potential baseline mental health moderators included the Generalized Anxiety Disorder Scale (7 items) [16]; the Brief Resilience Scale (6 items) [17]; the Perceived Stress Scale (10 items) [18]; the Patient Health Questionnaire (8 items, maximum 24-point score) and the moderate-or-higher-depression-severity classification (>10 on Patient Health Questionnaire) [19].

### 2.3. Statistical Analyses

The effect of moderation was tested with an interaction term between the intervention and potential moderator on the difference in change from 0 to 24 weeks between groups using linear, mixed-effects, multilevel models. These models included random cluster (community) effects to account for community-level randomization and correlation between participants in the same community. The covariates in the models determined a priori included age and education. Intention-to-treat analyses that included all the participants as randomized were conducted, regardless of the number of assessments performed or intervention attendance. For this analysis, one-sided tests were used to explore the impact of moderators on improvements in each outcome (e.g., weight loss) with a 95% confidence interval (95%CI). While two-sided tests are often automatically used, one-sided tests are warranted when the direction of the outcome is predetermined (e.g., improvements) and is more liberal in identifying potential moderators, which is of importance for not missing the potential moderators that could impact intervention refinement and dissemination [20]. Complete case analysis was used with a restricted maximum likelihood and incorporation of all the available data. For the outcome variables, no data were missing at the baseline; 50 participants (27%) withdrew or did not complete the data collection visit at 24 weeks. For the moderators, the degree of missingness was 5% at the baseline and 37% at 24 weeks. All analyses were conducted in 2023–2024 using SAS, version 9.4. Dr. Rebecca A. Seguin-Fowler, as this study’s Principal Investigator, takes responsibility for integrity of the data and data analysis.

## 3. Results

**Figure 1:**
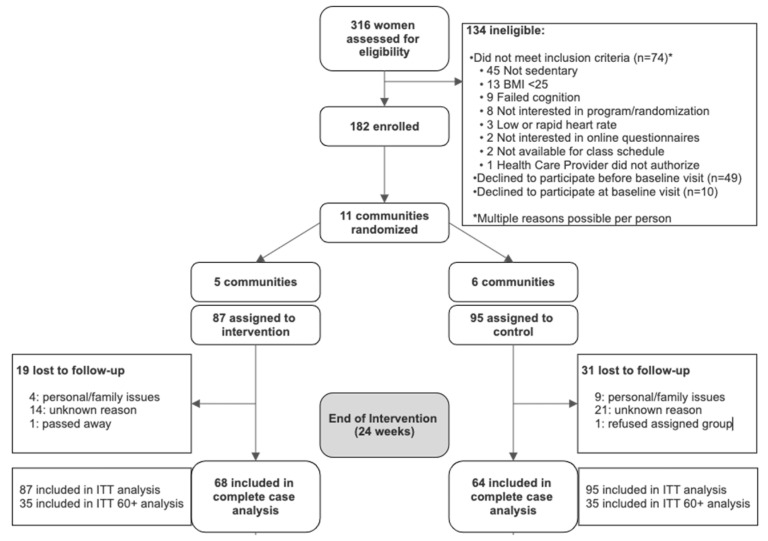
Profile for Strong Hearts, Healthy Communities-2.0 randomized trial.

Five communities were randomized into the intervention group (n = 87 participants), and six communities were randomized to the delayed intervention control group (n = 95 participants) (see Figure 1). The baseline characteristics for the total sample and each group are presented in Table 1.

### Moderation Effects

The moderation effects of the sociodemographic information and the baseline physical and mental health on weight loss, systolic blood pressure reduction, HbA1c reduction, and triglyceride reduction are presented in Table 2.

*Weight loss.* Greater weight loss was observed for the participants who were educated at the level of high school or less compared to those who had some post-high school education (−10.04 kg, 95% CI from −13.63 to −6.44); there were no significant moderation effects for any other sociodemographic potential moderator. Depression was also associated with differential weight loss, as those with higher baseline depression symptomatology demonstrated greater weight loss (−0.52 kg, 95%CI from −0.82 to −0.22), and those with moderate or higher depression severity had a weight loss of 6.45 kg greater than those with mild-to-no depression (95%CI from −10.30 to −2.60).

*Systolic blood pressure*. The participants who had a history of hypertension had a greater reduction in systolic blood pressure compared to those who did not (−7.71 mmHg, 95%CI from −13.95 to −1.47). No other moderator had a significant effect on systolic blood pressure.

*Hemoglobin A1c.* For the reduction in HbA1c, greater improvements were seen for the participants who had a history of cancer compared to those who did not (−0.28%, 95%CI from −0.55 to −0.02) and baseline moderate or higher depression severity compared to those who did not (−0.31%, 95%CI from −0.57 to −0.05).

*Triglycerides.* The sociodemographic characteristics associated with greater reductions in triglycerides included the participants who were employed (−53.49 mg/dL, 95%CI from −72.93 to −34.04) and those in a relationship (−32.49 mg/dL, 95%CI from −50.76 to −14.22). Across the health status moderators, the participants with a history of cancer also demonstrated a greater reduction in triglycerides (−48.72 mg/dL, 95%CI from −80.61 to −16.83). Higher health-related quality-of-life (−0.67 mg/dL, 95%CI from −1.12 to −0.22) and resilience scores (−36.00 mg/dL, 95%CI from −50.90 to −21.09) were associated with greater improvements in the level of triglycerides.

## 4. Discussion

In our analysis, the majority of the moderators evaluated did not have a significant effect on the health outcomes. As noted, most published papers on multilevel interventions did not evaluate the potential moderators of intervention effectiveness; thus, our findings have the potential to not only improve the future dissemination of the SHHC-2.0 intervention, but can also more broadly inform the development and evaluation of other multilevel interventions. While many sociodemographic characteristics were not associated with differential outcomes, we did find that those with lower educational attainment experienced greater weight loss; our data also showed that being employed and being in a relationship were associated with greater improvements in the level of triglycerides. Depression status was found to be a significant moderator for the primary outcome of weight loss, as the participants with worse depressive symptoms demonstrated greater weight loss. Higher depressive symptomatology was also associated with greater improvements in the HbA1c levels.

Women with moderate or higher depression severity at the baseline saw greater improvements in weight loss and reductions in the HbA1c level compared to those of their counterparts. Furthermore, similar trends were observed for systolic blood pressure and triglycerides, though these did not reach significance (*p* = 0.06). This finding is particularly promising given the complex and bi-directional relationship between weight and mental health. In a meta-analysis of longitudinal studies, there was an increased risk of depression for people who are overweight (27% increased risk) or obese (55%), while baseline depression increased the risk of the development of obesity by 58% [21]. The effect of weight loss interventions among persons with depression is unclear. Legenbauer et al. reported persons with depression had no differences in weight loss following a behavioral weight loss intervention [22]. However, other studies have reported poor weight loss outcomes among those with depression. For example, in a study of rural black women, depression was associated with less weight loss following a behavioral weight loss program [23]. The results of the current analysis suggests that the effectiveness of the SHHC 2.0 intervention is not impacted by depression status.

The prior studies have also identified depression status as negatively impacting behavior changes and the adherence to behavior change interventions [24,25,26,27,28]. To that end, we explored if women with elevated depressive symptoms at the baseline engaged with the intervention differently via attendance and intervention tool use (e.g., health journal, participant binder, Fitbit, digital scale, or exercise DVDs) and found no significant differences (results not reported). We could posit that there may not be a single facet of the intervention that was most impactful for these women, but in fact the multi-component, multilevel approach may have addressed the various needs of these women. However, these exploratory analyses were limited by a small sample size of women with baseline moderate or higher depression severity at the baseline (n = 9). One relevant finding for this subgroup is that 77% (n = 7) of the women who were classified as having a moderate or higher depression severity at baseline no longer met that categorization at the end of the intervention. This is in line with prior research indicating that weight loss can treat depressive symptoms, whether weight loss is achieved from surgery or through behavioral changes [29,30,31], and that changes in physical activity and diet have also shown to improve depressive symptoms [32,33].

Our results indicated that the participants with lower educational attainment saw greater weight loss, while being employed was associated with a greater improvement in triglycerides. The findings from a systematic review on how different aspects of inequality, including education and employment, impact uptake, adherence, and the effectiveness of trials of behavioral weight management interventions, suggest that education can have varying impacts on weight loss [34]. Lower educational attainment has been consistently linked to lower nutrition knowledge [35,36], and nutrition knowledge is associated with poorer diet quality [37]. It is possible that the SHHC-2.0 intervention is more effective in reducing weight in those with a high school education or less through improved health knowledge. The moderating effect of employment on triglycerides may reflect structural barriers experienced by those with fewer resources (e.g., participants with no employment may need the additional social or environmental supports), highlighting a potential opportunity to address and mitigate the social determinants of health for participants in the context of these types of programs.

The participants that reported being in a relationship also demonstrated greater improvements in the level of triglycerides. While the SHHC 2.0 intervention aimed to increase social support both among the participants and with friends and family, it is possible that more focused strategies to increase social support within and beyond the class setting and from a range of social network members (e.g., coworkers and faith-based community members) might be particularly beneficial for those not in a relationship.

While the moderators discussed above warrant further research, we note that most of the factors evaluated did not have a significant effect, or the effects were not consistent across all the health outcomes. The lack of heterogeneity in treatment responses across the subgroups may be due to the strong evidence base of the program [38], extensive tailoring via multiple rounds of formative research, and subsequent intervention refinement via process evaluations [39,40]. Additionally, health improvement is not one-size-fits-all, and having a multi-component (focused on diet and physical activity), multilevel (focused on individual behavior change, social support, and environmental changes) intervention creates multiple avenues for behavior changes and health improvements. This likely minimized the heterogeneity of intervention impact across the participant characteristics.

There are limitations to our inferences due to the exploratory nature of this analysis. The original study was not powered for moderation analysis; thus, null findings may be due to a lack of power resulting from small subgroups, while significant findings could be the result of small comparison groups. In part, that is why we interpreted our findings with one-sided testing with established improvements in the outcomes (e.g., weight loss) to be more liberal in identifying the potential moderators that could impact intervention refinement and dissemination. Even so, the consistency and lack of moderation in the outcomes may also be due to the rural, medically underserved towns and participants, reflecting their predominantly White populations. Future studies in more racially and ethnically diverse communities are needed to truly assess the consistency in outcomes observed with SHHC 2.0. Furthermore, it is possible that the other factors not assessed in this study (e.g., community-level factors, sleep, and health beliefs/attitudes) could moderate the intervention effects. The rates of missing data may also impact our findings. At 24 weeks, 50 participants (27%) withdrew or did not complete the data collection visit, so it is possible that the missing data could impact the reliability of our results. The primary concern was that data may not be missing at random and that participants with worse health might be more likely to drop out or not report. To explore this potential bias, the baseline characteristics of the respondents and the non-respondents were compared at 24 weeks. No significant differences were observed for age, income, education, race, BMI, weight, physical activity levels, or self-reported perceived overall health. These findings do not indicate systemic bias resulting from missing data, though we note that unobserved differences between the groups may exist. We chose to report this complete case analysis rather than conduct multiple imputation (MI) given the exploratory nature of this report, which did not require more representative parameter estimates (a strength of MI). Future studies with larger samples would benefit from exploring non-linear moderation effects as well. The strengths include exploring multiple health outcomes (e.g., weight loss, reduction in systolic blood pressure, reduction in HbA1c, and reduction in triglycerides) and a variety of moderators, specifically the inclusion of baseline mental health.

## 5. Conclusions

SHHC is effective [11,12] and cost-effective [41], and based on these findings, likely beneficial for a wide range of participants, making it well poised for wider dissemination and impacts. These findings are critical for intervention-specific dissemination and as a demonstration for future interventions to critically examine the impacts across participant characteristics to ensure interventions are optimizing the opportunities to help address health equity. Further efforts to broaden the dissemination of the SHHC-2.0 intervention could also include adaptations for other populations (i.e., women living in urban/suburban areas), or alternative delivery strategies (i.e., digital or hybrid intervention delivery). Another area for future research is related to the maintenance of the observed effects. We note that the SHHC-2.0 intervention effects were maintained over a 6-month follow-up period [12], but studies with a longer follow-up period are needed to demonstrate the long-term maintenance of the intervention effects. Finally, the SHHC-2.0 intervention may have positive health impacts beyond those evaluated in the current analysis (i.e., mental health improvements) that can be evaluated in future studies.

## Figures and Tables

**Table 1:** Strong Hearts, Healthy Communities-2.0 participant characteristics at baseline.

	Total (n = 182)	Control (n = 95)	Intervention (n = 87)
Age (n = 182), years ±SD	57.2 ± 9.0	55.9 ± 8.5	58.5 ± 9.3
Employment (n = 130), n (%)			
Working	120 (92.3)	62 (92.5)	85 (92.1)
Not working	10 (7.7)	5 (7.5)	5 (7.9)
Income, household annual (n = 162), n (%)			
<USD 50,000	66 (40.7)	32 (19.7)	34 (21.0)
≥USD 50,000	96 (59.3)	130 (80.3)	128 (79.0)
Education (n = 172), n (%)			
High school or less	35 (20.3)	17 (19.5)	18 (21.2)
Some college or more	137 (79.7)	70 (80.5)	67 (78.8)
Relationship status (n = 171), n (%)			
In a relationship	116 (67.8)	62 (71.3)	54 (64.3)
Not in a relationship	55 (32.2)	25 (28.7)	30 (35.7)
Body Mass Index, kg/m^2^ (n = 182), mean ±SD	36.7 ± 7.8	37.9 ± 8.5	35.4 ± 6.8
History of arthritis (n = 170), n (%)	70 (41.2)	39 (44.8)	31 (37.3)
History of cancer (n = 170), n (%)	12 (7.1)	5 (5.7)	7 (8.4)
History of diabetes (n = 170), n (%)	25 (14.7)	17 (19.5)	8 (9.6)
History of heart disease (n = 170), n (%)	10 (5.9)	5 (5.7)	5 (6.0)
History of high blood cholesterol (n = 170), n (%)	71 (41.8)	33 (38.4)	38 (45.2)
History of high blood sugar (n = 170), n (%)	37 (21.8)	16 (19.3)	21 (24.1)
History of hypertension (n = 170), n (%)	71 (41.8)	41 (47.7)	30 (35.7)
Short Form health-related quality of life (SF36) (n = 171), 36 items	81.6 ± 20.1	81.0 ± 20.8	82.3 ± 19.4
Generalized Anxiety Disorder Scale (n = 171), 7 items	2.6 ± 3.3	2.8 ± 3.3	2.4 ± 3.3
Brief Resilience Scale (n = 171), 6 items	3.8 ± 0.6	3.9 ± 0.6	3.7 ± 0.7
Perceived Stress Scale (n = 170), 10 items	4.8 ± 3.0	4.9 ± 3.2	4.8 ± 3.2
Patient Health Questionnaire (Depression) (n = 171), 8 items	4.4 ± 3.9	5.0 ± 4.0	3.9 ± 3.8
Moderate or higher depression severity (n = 171), (≥10 on Patient Health Questionnaire)	19 (11.1)	11 (12.6)	8 (9.5)

Notes: For dichotomous variables, count and % of each sample are reported for level listed (e.g., employment, working). For continuous variables, mean ± SD is reported.

**Table 2:** Moderation effects of between-group changes in outcomes from baseline to intervention end point (24 weeks).

	Weight Loss (kg)		Reduction in Systolic Blood Pressure (mmHg)		Reduction in Hemoglobin A1c (%)		Reduction in Triglycerides (mg/dL) Potential Moderators	
	**n**	**Est (95% CI) *p*-Value**	**n**	**Est (95% CI) *p*-Value**	**n**	**Est (95% CI) *p*-Value**	**n**	**Est (95% CI) *p*-Value**
Age, years	131	−0.08 (−0.20, 0.03) *p* = 0.121	130	0.05 (−0.31, 0.40) *p* = 0.587	130	0.00 (0.00, 0.01) *p* = 0.799	129	1.45 (0.49, 2.41) ^a^ *p* = 0.993
Employment, working	97	3.37 (0.97, 5.76) ^a^ *p* = 0.989	96	−0.83 (−8.07, 6.41) *p* = 0.425	98	−0.10 (−0.26, 0.06) *p* = 0.145	98	**−53.49 (−72.93, −34.04) *p* < 0.001**
Income, ≤USD 50 k	121	1.04 (−3.51, 5.60) *p* = 0.354	120	0.55 (−0.96, 2.06) *p* = 0.724	120	0.02 (−0.26, 0.29) *p* = 0.539	119	−13.48 (−46.33, 19.38) *p* = 0.251
Education, high school or less	129	**−10.04 (−13.63, −6.44) *p* < 0.001**	128	−4.85 (−16.49, 6.79) *p* = 0.248	128	0.07 (−0.18, 0.32) *p* = 0.676	127	21.12 (−9.57, 51.80) *p* = 0.870
In a relationship status	128	2.52 (0.27, 4.77) ^a^ *p* = 0.966	127	2.23 (−4.49, 8.95) *p* = 0.707	127	0.11 (−0.04, 0.25) *p* = 0.880	126	**−32.49 (−50.76, −14.22) *p* = 0.002**
Body Mass Index, kg/m^2^	131	0.03 (−0.11, 0.17) *p* = 0.623	130	0.13 (−0.29, 0.55) *p* = 0.692	130	−0.01 (−0.02, 0.00) *p* = 0.092	129	1.15 (0.01, 2.30) ^a^ *p* = 0.950
History of arthritis	127	1.41 (−0.84, 3.67) *p* = 0.848	126	−5.09 (−11.61, 1.43) *p* = 0.101	126	−0.12 (−0.27, 0.03) *p* = 0.089	125	0.20 (−17.88, 18.27) *p* = 0.507
History of cancer	127	2.32 (−1.77, 6.40) *p* = 0.824	126	−4.76 (−16.31, 6.78) *p* = 0.250	126	**−0.28 (−0.55, −0.02) *p* = 0.041**	125	**−48.72 (−80.61, −16.83) *p* = 0.006**
History of diabetes	127	−1.51 (−5.21, 2.18) *p* = 0.252	126	7.59 (−3.01, 18.19) *p* = 0.879	126	0.76 (0.54, 0.99) ^a^ *p* = 1.000	125	22.89 (−4.78, 50.56) *p* = 0.912
History of heart disease	127	−1.60 (−6.50, 3.31) *p* = 0.297	126	−12.53 (−26.53, 1.46) *p* = 0.072	126	0.30 (−0.03, 0.62) *p* = 0.934	125	21.35 (−17.43, 60.13) *p* = 0.816
History of high blood cholesterol	127	2.00 (−0.21, 4.22) *p* = 0.930	126	3.85 (−2.43, 10.13) *p* = 0.842	126	0.01 (−0.14, 0.16) *p* = 0.545	125	−1.69 (−20.01, 16.63) *p* = 0.440
History of high blood sugar	127	−2.31 (−4.96, 0.35) *p* = 0.078	126	6.9 (−0.81, 14.62) *p* = 0.928	126	0.25 (0.08, 0.43) ^a^ *p* = 0.991	125	−17.30 (−38.21, 3.61) *p* = 0.088
History of hypertension	127	0.17 (−2.06, 2.39) *p* = 0.549	126	**−7.71 (−13.95, −1.47) *p* = 0.022**	126	0.16 (0.01, 0.31) ^a^ *p* = 0.958	125	−11.54 (−30.00, 6.93) *p* = 0.153
Short Form health-related quality of life, 36 items	128	−0.02 (−0.07, 0.03) *p* = 0.286	127	−0.04 (−0.19, 0.11) *p* = 0.335	127	0.00 (0.00, 0.00) *p* = 0.284	126	**−0.67 (−1.12, −0.22) *p* = 0.008** Generalized Anxiety Scale, 7 items
Generalized Anxiety Scale, 7 items	128	−0.22 (−0.56, 0.12) *p* = 0.142	127	0.07 (−0.87, 1.01) *p* = 0.549	127	−0.02 (−0.04, 0.00) *p* = 0.085	126	0.73 (−1.98, 3.43) *p* = 0.670
Brief Resilience Scale, 6 items	128	0.07 (−1.78, 1.92) *p* = 0.525	127	4.12 (−1.24, 9.47) *p* = 0.896	127	−0.07 (−0.19, 0.05) *p* = 0.181	126	**−36.00 (−50.90, −21.09) *p* < 0.001**
Perceived Stress Scale, 10 items	127	−0.04 (−0.24, 0.16) *p* = 0.376	126	−0.16 (−0.74, 0.42) *p* = 0.326	126	0.00 (−0.02, 0.01) *p* = 0.394	125	2.33 (0.71, 3.94) ^a^ *p* = 0.991
Patient Health Questionnaire—8 items	128	**−0.52 (−0.82, −0.22) *p* = 0.002**	127	−2.75 (−7.48, 1.99) *p* = 0.171	127	−0.02 (−0.04, 0) *p* = 0.105	126	2.06 (−0.46, 4.59) *p* = 0.909
Moderate or higher depression severity	128	**−6.45 (−10.30, −2.60) *p* = 0.003**	127	−10.19 (−21.41, 1.03) *p* = 0.069	127	**−0.31 (−0.57, −0.05) *p* = 0.024**	**126**	−8.27 (−17.09, 0.55) *p* = 0.063

Notes: All estimates are from complete case models and adjusted for random cluster (community) effects, assignment group, age, and education. Confidence intervals and *p*-values are adjusted for one-sided tests on effect of moderator on outcomes of weight loss, reduction in systolic blood pressure, reduction in hemoglobin A1c, and reduction in triglycerides. ^a^ indicates 95%CI that is in opposite direction of one-sided test. **Bold** indicates *p* value < 0.05.

## Data Availability

The data presented in this study are available on request from the corresponding author. The data are not publicly available due to use of human subject data.
