# Correction: Rethorst et al. Heterogeneity in Health Outcomes in the Strong Hearts, Healthy Communities-2.0 Multilevel Intervention in a Community-Randomized Trial: An Exploratory Study of Moderators. *Nutrients* 2024, *16*, 4353

**DOI:** 10.3390/nu18050728

**Published:** 2026-02-25

**Authors:** Chad D. Rethorst, Margaret M. Demment, Seungyeon Ha, Sara C. Folta, Meredith L. Graham, Galen D. Eldridge, Rebecca A. Seguin-Fowler

**Affiliations:** 1 Institute for Advancing Health Through Agriculture, Texas A&M University, Dallas, TX 75252, USA; chad.rethorst@ag.tamu.edu (C.D.R.); margaret.demment@ag.tamu.edu (M.M.D.); meredith.graham@ag.tamu.edu (M.L.G.); galen.eldridge@ag.tamu.edu (G.D.E.); 2 Statistical Consultation Center, Texas A&M University, College Station, TX 77843, USA; ha.shawn@tamu.edu; 3 Friedman School of Nutrition Science and Policy, Tufts University, Boston, MA 02111, USA; sara.folta@tufts.edu; 4 Institute for Advancing Health Through Agriculture, Texas A&M University, College Station, TX 77840, USA

**Text Correction**

There was an error in the original publication [1]. There was a misinterpretation of the output of the dichotomous education variable.

A correction has been made to the abstract. The corrected abstract is below.

**Abstract:** Background/Objectives: Multilevel interventions have demonstrated efficacy in improving obesity and other related health outcomes. However, heterogeneity in individual responses indicates the need to identify the factors associated with responses and non-responses to multilevel interventions. The objective of this report is to identify the potential sources of heterogeneity through the exploration of the moderation effects of participant characteristics (sociodemographic and baseline physical/mental health) in the Strong Hearts, Healthy Communities-2.0 (SHHC-2.0) intervention. Methods: SHHC-2.0 is a 24-week multilevel intervention to improve people’s diet and physical activity evaluated using a cluster-randomized, controlled trial design conducted with women aged 40 and older living in rural communities with an elevated risk of cardiovascular disease, defined as having a BMI > 30, or a BMI 25–30 plus < 1 weekly occurrence of 30 min of physical activity during leisure time. Linear mixed models were used to compare the between-group changes in the outcomes (weight, systolic blood pressure, hemoglobin A1c [HbA1c], and triglycerides), with an interaction term included for each potential moderator. Results: Within the sociodemographic characteristics, there were no differences in effectiveness by age, income, or baseline BMI status, however the participants with a high school education or less experienced greater weight loss. Among their health history, only a history of hypertension was associated with differential outcomes; those with a history of hypertension demonstrated a greater reduction in systolic blood pressure. The participants with elevated depressive symptoms demonstrated greater weight loss and a greater reduction in the HbA1c level. Conclusions: SHHC-2.0 was effective across a wide range of participants. The identified moderators (i.e., education level) may inform the future tailoring of the SHHC intervention to optimize the outcomes among participant subgroups, while more broadly, our findings can serve to inform the development and dissemination of multilevel interventions.

A correction has been made to 3. Results, Moderation Effects, Weight loss. The corrected paragraph is below.

Greater weight loss was observed for the participants who were educated at the level of high school or less compared to those who had some post-high school education (−10.04 kg, 95% CI from −13.63 to −6.44); there were no significant moderation effects for any other sociodemographic potential moderator. Depression was also associated with differential weight loss, as those with higher baseline depression symptomatology demonstrated greater weight loss (−0.52 kg, 95%CI from −0.82 to −0.22), and those with moderate or higher depression severity had a weight loss of 6.45 kg greater than those with mild-to-no depression (95%CI from −10.30 to −2.60).

A correction has been made to 4. Discussion, Paragraph 1. The corrected paragraph is below.

In our analysis, the majority of the moderators evaluated did not have a significant effect on the health outcomes. As noted, most published papers on multilevel interventions did not evaluate the potential moderators of intervention effectiveness; thus, our findings have the potential to not only improve the future dissemination of the SHHC-2.0 intervention, but can also more broadly inform the development and evaluation of other multilevel interventions. While many sociodemographic characteristics were not associated with differential outcomes, we did find that those with lower educational attainment experienced greater weight loss; our data also showed that being employed and being in a relationship were associated with greater improvements in the level of triglycerides. Depression status was found to be a significant moderator for the primary outcome of weight loss, as the participants with worse depressive symptoms demonstrated greater weight loss. Higher depressive symptomatology was also associated with greater improvements in the HbA1c levels.

A correction has been made to 4. Discussion, Paragraph 4. The corrected paragraph is below.

Our results indicated that the participants with lower educational attainment saw greater weight loss, while being employed was associated with a greater improvement in triglycerides. The findings from a systematic review on how different aspects of inequality, including education and employment, impact uptake, adherence, and the effectiveness of trials of behavioral weight management interventions, suggest that education can have varying impacts on weight loss [34]. Lower educational attainment has been consistently linked to lower nutrition knowledge [35,36], and nutrition knowledge is associated with poorer diet quality [37]. It is possible that the SHHC-2.0 intervention is more effective in reducing weight in those with a high school education or less through improved health knowledge. The moderating effect of employment on triglycerides may reflect structural barriers experienced by those with fewer resources (e.g., participants with no employment may need the additional social or environmental supports), highlighting a potential opportunity to address and mitigate the social determinants of health for participants in the context of these types of programs.

A correction has been made to 4. Discussion, Paragraph 6. The corrected paragraph is below.

While the moderators discussed above warrant further research, we note that most of the factors evaluated did not have a significant effect, or the effects were not consistent across all the health outcomes. The lack of heterogeneity in treatment responses across the subgroups may be due to the strong evidence base of the program [38], extensive tailoring via multiple rounds of formative research, and subsequent intervention refinement via process evaluations [39,40]. Additionally, health improvement is not one-size-fits-all, and having a multi-component (focused on diet and physical activity), multilevel (focused on individual behavior change, social support, and environmental changes) intervention creates multiple avenues for behavior changes and health improvements. This likely minimized the heterogeneity of intervention impact across the participant characteristics.

A correction has been made to 5. Conclusions. The corrected paragraph is below.

SHHC is effective [11,12] and cost-effective [41], and based on these findings, likely beneficial for a wide range of participants, making it well poised for wider dissemination and impacts. These findings are critical for intervention-specific dissemination and as a demonstration for future interventions to critically examine the impacts across participant characteristics to ensure interventions are optimizing the opportunities to help address health equity. Further efforts to broaden the dissemination of the SHHC-2.0 intervention could also include adaptations for other populations (i.e., women living in urban/suburban areas), or alternative delivery strategies (i.e., digital or hybrid intervention delivery). Another area for future research is related to the maintenance of the observed effects. We note that the SHHC-2.0 intervention effects were maintained over a 6-month follow-up period [12], but studies with a longer follow-up period are needed to demonstrate the long-term maintenance of the intervention effects. Finally, the SHHC-2.0 intervention may have positive health impacts beyond those evaluated in the current analysis (i.e., mental health improvements) that can be evaluated in future studies.

**Tables Corrections**

In Table 1, the Education label should not have ‘some college or more’ included as part of the label. In Table 2, the Education label should say ‘Education, high school or less’ instead of ‘Education, some college or more.’ The corrected Table 1 and Table 2 appear below. The original publication has also been updated.

**References**

With this correction, the order of some references has been adjusted accordingly. The original publication has also been updated. The newly added reference list is below.

35. Koch, F.; Hoffmann, I.; Claupein, E. Types of nutrition knowledge, their socio-demographic determinants and their association with food consumption: Results of the NEMONIT study. *Front. Nutr.* **2021**, *8*, 630014.
36. Parmenter, K.; Waller, J.; Wardle, J. Demographic variation in nutrition knowledge in England. *Health Educ. Res.* **2000**, *15*, 163–174.
37. Spronk, I.; Kullen, C.; Burdon, C.; O’Connor, H. Relationship between nutrition knowledge and dietary intake. *Br. J. Nutr.* **2014**, *111*, 1713–1726.

The authors state that the other scientific conclusions are unaffected. These corrections were approved by the Academic Editor. The original publication has also been updated.

## Figures and Tables

**Table 1:** Strong Hearts, Healthy Communities-2.0 participant characteristics at baseline.

	Total (n = 182)	Control (n = 95)	Intervention (n = 87)
Age (n = 182), years ±SD	57.2 ± 9.0	55.9 ± 8.5	58.5 ± 9.3
Employment (n = 130), n (%)			
Working	120 (92.3)	62 (92.5)	85 (92.1)
Not working	10 (7.7)	5 (7.5)	5 (7.9)
Income, household annual (n = 162), n (%)			
<USD 50,000	66 (40.7)	32 (19.7)	34 (21.0)
≥USD 50,000	96 (59.3)	130 (80.3)	128 (79.0)
Education (n = 172), n (%)			
High school or less	35 (20.3)	17 (19.5)	18 (21.2)
Some college or more	137 (79.7)	70 (80.5)	67 (78.8)
Relationship status (n = 171), n (%)			
In a relationship	116 (67.8)	62 (71.3)	54 (64.3)
Not in a relationship	55 (32.2)	25 (28.7)	30 (35.7)
Body Mass Index, kg/m^2^ (n = 182), mean ±SD	36.7 ± 7.8	37.9 ± 8.5	35.4 ± 6.8
History of arthritis (n = 170), n (%)	70 (41.2)	39 (44.8)	31 (37.3)
History of cancer (n = 170), n (%)	12 (7.1)	5 (5.7)	7 (8.4)
History of diabetes (n = 170), n (%)	25 (14.7)	17 (19.5)	8 (9.6)
History of heart disease (n = 170), n (%)	10 (5.9)	5 (5.7)	5 (6.0)
History of high blood cholesterol (n = 170), n (%)	71 (41.8)	33 (38.4)	38 (45.2)
History of high blood sugar (n = 170), n (%)	37 (21.8)	16 (19.3)	21 (24.1)
History of hypertension (n = 170), n (%)	71 (41.8)	41 (47.7)	30 (35.7)
Short Form health-related quality of life (SF36) (n = 171), 36 items	81.6 ± 20.1	81.0 ± 20.8	82.3 ± 19.4
Generalized Anxiety Disorder Scale (n = 171), 7 items	2.6 ± 3.3	2.8 ± 3.3	2.4 ± 3.3
Brief Resilience Scale (n = 171), 6 items	3.8 ± 0.6	3.9 ± 0.6	3.7 ± 0.7
Perceived Stress Scale (n = 170), 10 items	4.8 ± 3.0	4.9 ± 3.2	4.8 ± 3.2
Patient Health Questionnaire (Depression) (n = 171), 8 items	4.4 ± 3.9	5.0 ± 4.0	3.9 ± 3.8
Moderate or higher depression severity (n = 171), (≥10 on Patient Health Questionnaire)	19 (11.1)	11 (12.6)	8 (9.5)

Notes: For dichotomous variables, count and % of each sample are reported for level listed (e.g., employment, working). For continuous variables, mean ± SD is reported.

**Table 2:** Moderation effects of between-group changes in outcomes from baseline to intervention end point (24 weeks).

	Weight Loss (kg)		Reduction in Systolic Blood Pressure (mmHg)		Reduction in Hemoglobin A1c (%)		Reduction in Triglycerides (mg/dL) Potential Moderators	
	**n**	**Est (95% CI) *p*-Value**	**n**	**Est (95% CI) *p*-Value**	**n**	**Est (95% CI) *p*-Value**	**n**	**Est (95% CI) *p*-Value**
Age, years	131	−0.08 (−0.20, 0.03) *p* = 0.121	130	0.05 (−0.31, 0.40) *p* = 0.587	130	0.00 (0.00, 0.01) *p* = 0.799	129	1.45 (0.49, 2.41) ^a^ *p* = 0.993
Employment, working	97	3.37 (0.97, 5.76) ^a^ *p* = 0.989	96	−0.83 (−8.07, 6.41) *p* = 0.425	98	−0.10 (−0.26, 0.06) *p* = 0.145	98	**−53.49 (−72.93, −34.04) *p* < 0.001**
Income, ≤USD 50 k	121	1.04 (−3.51, 5.60) *p* = 0.354	120	0.55 (−0.96, 2.06) *p* = 0.724	120	0.02 (−0.26, 0.29) *p* = 0.539	119	−13.48 (−46.33, 19.38) *p* = 0.251
Education, high school or less	129	**−10.04 (−13.63, −6.44) *p* < 0.001**	128	−4.85 (−16.49, 6.79) *p* = 0.248	128	0.07 (−0.18, 0.32) *p* = 0.676	127	21.12 (−9.57, 51.80) *p* = 0.870
In a relationship status	128	2.52 (0.27, 4.77) ^a^ *p* = 0.966	127	2.23 (−4.49, 8.95) *p* = 0.707	127	0.11 (−0.04, 0.25) *p* = 0.880	126	**−32.49 (−50.76, −14.22) *p* = 0.002**
Body Mass Index, kg/m^2^	131	0.03 (−0.11, 0.17) *p* = 0.623	130	0.13 (−0.29, 0.55) *p* = 0.692	130	−0.01 (−0.02, 0.00) *p* = 0.092	129	1.15 (0.01, 2.30) ^a^ *p* = 0.950
History of arthritis	127	1.41 (−0.84, 3.67) *p* = 0.848	126	−5.09 (−11.61, 1.43) *p* = 0.101	126	−0.12 (−0.27, 0.03) *p* = 0.089	125	0.20 (−17.88, 18.27) *p* = 0.507
History of cancer	127	2.32 (−1.77, 6.40) *p* = 0.824	126	−4.76 (−16.31, 6.78) *p* = 0.250	126	**−0.28 (−0.55, −0.02) *p* = 0.041**	125	**−48.72 (−80.61, −16.83) *p* = 0.006**
History of diabetes	127	−1.51 (−5.21, 2.18) *p* = 0.252	126	7.59 (−3.01, 18.19) *p* = 0.879	126	0.76 (0.54, 0.99) ^a^ *p* = 1.000	125	22.89 (−4.78, 50.56) *p* = 0.912
History of heart disease	127	−1.60 (−6.50, 3.31) *p* = 0.297	126	−12.53 (−26.53, 1.46) *p* = 0.072	126	0.30 (−0.03, 0.62) *p* = 0.934	125	21.35 (−17.43, 60.13) *p* = 0.816
History of high blood cholesterol	127	2.00 (−0.21, 4.22) *p* = 0.930	126	3.85 (−2.43, 10.13) *p* = 0.842	126	0.01 (−0.14, 0.16) *p* = 0.545	125	−1.69 (−20.01, 16.63) *p* = 0.440
History of high blood sugar	127	−2.31 (−4.96, 0.35) *p* = 0.078	126	6.9 (−0.81, 14.62) *p* = 0.928	126	0.25 (0.08, 0.43) ^a^ *p* = 0.991	125	−17.30 (−38.21, 3.61) *p* = 0.088
History of hypertension	127	0.17 (−2.06, 2.39) *p* = 0.549	126	**−7.71 (−13.95, −1.47) *p* = 0.022**	126	0.16 (0.01, 0.31) ^a^ *p* = 0.958	125	−11.54 (−30.00, 6.93) *p* = 0.153
Short Form health-related quality of life, 36 items	128	−0.02 (−0.07, 0.03) *p* = 0.286	127	−0.04 (−0.19, 0.11) *p* = 0.335	127	0.00 (0.00, 0.00) *p* = 0.284	126	**−0.67 (−1.12, −0.22) *p* = 0.008** Generalized Anxiety Scale, 7 items
Generalized Anxiety Scale, 7 items	128	−0.22 (−0.56, 0.12) *p* = 0.142	127	0.07 (−0.87, 1.01) *p* = 0.549	127	−0.02 (−0.04, 0.00) *p* = 0.085	126	0.73 (−1.98, 3.43) *p* = 0.670
Brief Resilience Scale, 6 items	128	0.07 (−1.78, 1.92) *p* = 0.525	127	4.12 (−1.24, 9.47) *p* = 0.896	127	−0.07 (−0.19, 0.05) *p* = 0.181	126	**−36.00 (−50.90, −21.09) *p* < 0.001**
Perceived Stress Scale, 10 items	127	−0.04 (−0.24, 0.16) *p* = 0.376	126	−0.16 (−0.74, 0.42) *p* = 0.326	126	0.00 (−0.02, 0.01) *p* = 0.394	125	2.33 (0.71, 3.94) ^a^ *p* = 0.991
Patient Health Questionnaire—8 items	128	**−0.52 (−0.82, −0.22) *p* = 0.002**	127	−2.75 (−7.48, 1.99) *p* = 0.171	127	−0.02 (−0.04, 0) *p* = 0.105	126	2.06 (−0.46, 4.59) *p* = 0.909
Moderate or higher depression severity	128	**−6.45 (−10.30, −2.60) *p* = 0.003**	127	−10.19 (−21.41, 1.03) *p* = 0.069	127	**−0.31 (−0.57, −0.05) *p* = 0.024**	**126**	−8.27 (−17.09, 0.55) *p* = 0.063

Notes: All estimates are from complete case models and adjusted for random cluster (community) effects, assignment group, age, and education. Confidence intervals and *p*-values are adjusted for one-sided tests on effect of moderator on outcomes of weight loss, reduction in systolic blood pressure, reduction in hemoglobin A1c, and reduction in triglycerides. ^a^ indicates 95%CI that is in opposite direction of one-sided test. **Bold** indicates *p* value < 0.05.
